# 1-Hydroxy-4-aza-1-azoniabicyclo[2.2.2]octane picrate

**DOI:** 10.1107/S1600536810023597

**Published:** 2010-06-23

**Authors:** Jing-Mei Xiao

**Affiliations:** a Ordered Matter Science Research Center, College of Chemistry and Chemical Engineering, Southeast University, Nanjing 211189, People’s Republic of China

## Abstract

In the crystal structure of the title compound, C_6_H_13_N_2_O^+^·C_6_H_2_N_3_O_7_^−^, the anions and cations are linked by O—H⋯O, C—H⋯O and C—H⋯N hydrogen bonds into a three-dimensional network. The O atoms of a nitro group of the picrate anion are disordered over two positions of equal occupancy.

## Related literature

For the dielectric properties of *N*-protonated compounds, see: Szafranski & Katrusiak (2008 ▶); Katrusiak & Szafranski (1999 ▶); Chen *et al.* (2009 ▶); Mihailovic *et al.* (1990 ▶).
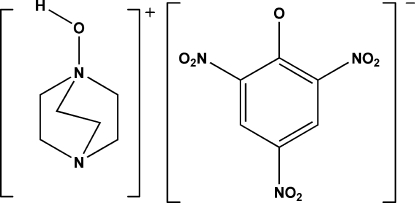


## Experimental

### 

#### Crystal data

C_6_H_13_N_2_O^+^·C_6_H_2_N_3_O_7_^−^
*M* *_r_* = 357.29
Monoclinic, 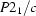

*a* = 11.521 (2) Å
*b* = 19.230 (4) Å
*c* = 6.9921 (14) Å
β = 98.73 (3)°
*V* = 1531.2 (5) Å^3^
*Z* = 4
Mo *K*α radiation
μ = 0.13 mm^−1^
*T* = 293 K
0.3 × 0.25 × 0.2 mm

#### Data collection

Rigaku Mercury2 diffractometer
Absorption correction: multi-scan (*CrystalClear*; Rigaku, 2005 ▶) *T*_min_ = 0.176, *T*_max_ = 0.298
15623 measured reflections
3495 independent reflections
2111 reflections with *I* > 2σ(*I*)
*R*_int_ = 0.064

#### Refinement

*R*[*F* ^2^ > 2σ(*F* ^2^)] = 0.056
*wR*(*F* ^2^) = 0.176
*S* = 0.83
3495 reflections
249 parameters
H atoms treated by a mixture of independent and constrained refinement
Δρ_max_ = 0.18 e Å^−3^
Δρ_min_ = −0.17 e Å^−3^

### 

Data collection: *CrystalClear* (Rigaku, 2005 ▶); cell refinement: *CrystalClear*; data reduction: *CrystalClear*; program(s) used to solve structure: *SHELXS97* (Sheldrick, 2008 ▶); program(s) used to refine structure: *SHELXL97* (Sheldrick, 2008 ▶); molecular graphics: *SHELXTL* (Sheldrick, 2008 ▶); software used to prepare material for publication: *SHELXL97*.

## Supplementary Material

Crystal structure: contains datablocks I, New_Global_Publ_Block. DOI: 10.1107/S1600536810023597/rz2465sup1.cif

Structure factors: contains datablocks I. DOI: 10.1107/S1600536810023597/rz2465Isup2.hkl

Additional supplementary materials:  crystallographic information; 3D view; checkCIF report

## Figures and Tables

**Table 1:** Hydrogen-bond geometry (Å, °)

*D*—H⋯*A*	*D*—H	H⋯*A*	*D*⋯*A*	*D*—H⋯*A*
O8—H1⋯O7	0.88 (3)	1.74 (4)	2.602 (3)	167 (3)
O8—H1⋯O6′	0.88 (3)	2.50 (4)	2.973 (15)	115 (3)
C7—H7*B*⋯O1	0.97	2.59	3.486 (4)	153
C9—H9*A*⋯O7	0.97	2.42	3.102 (3)	127
C5—H5⋯N5^i^	0.93	2.61	3.536 (3)	171
C11—H11*A*⋯O6′^ii^	0.97	2.54	3.342 (15)	139
C12—H12*A*⋯O5′^iii^	0.97	2.31	3.176 (9)	149
C12—H12*B*⋯O5^iv^	0.97	2.55	3.137 (7)	119

Symmetry codes: (i) 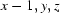; (ii) 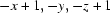; (iii) 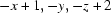; (iv) 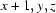.

**Fig. 1.**
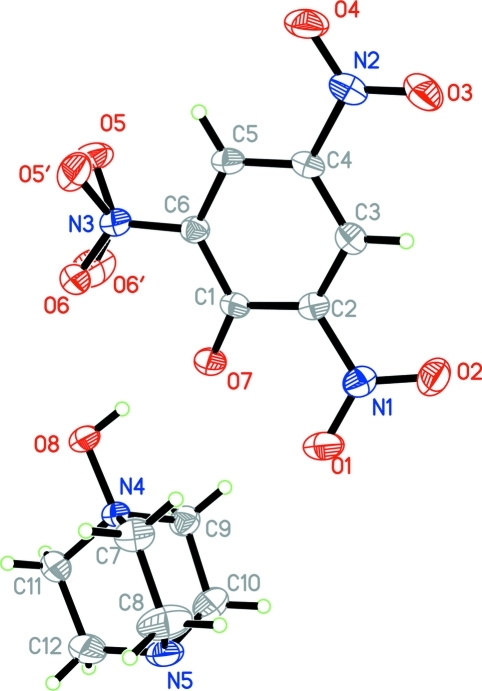
The molecular structure of the title compound, showing the atomic numbering scheme. Displacement ellipsoids are drawn at the 30% probability level.

**Fig. 2.**
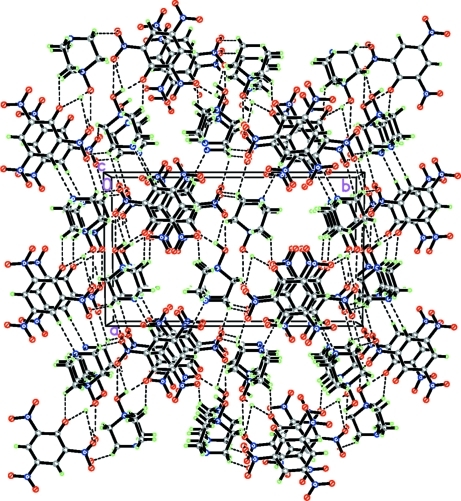
The crystal packing of the title compound, viewed along the c axis. Dashed lines indicate hydrogen bonds.

## supplementary crystallographic information

### Comment

The variable-temperature dielectric response, especially in relatively high frequency range, is very useful for searching phase transitions in which there is a dielectric anomaly at the transition temperature (Szafranski & Katrusiak, 2008; Katrusiak & Szafranski, 1999; Chen *et al.*, 2009; Mihailovic *et al.*, 1990). As part of a study on phase transitions of *N*-protonated compounds, the title compound has been synthesized and its dielectric properties measured. The title compound (m. p. = 405-410 K), however, showed no dielectric disuniformity in the range 93–395 K.

The asymmetric unit of the title compound (Fig. 1) contains one protonated triethylenediamine-*N*-oxide cation and one picrate anion. In the anion, the oxygen atoms of the nitro group including atom N3 are disordered over two positions with site occupancies of 0.5. The N1/O1/O2, N2/O3/O4, N3/O5/O6 and N3/O5'/O6' nitro groups are tilted with respect to the plane of the benzene ring by 31.6 (2), 4.8 (2), 23.8 (4) and 30.2 (6)° respectively. In the crystal structure (Fig. 2), anions and cations are linked by O—H···O, C—H···O and C—H···O hydrogen bonds (Table 1) into a three-dimensional network.

### Experimental

To a solution of triethylenediamine (50 mmol, 5.6 g) in benzene (500 ml) was added rapidly H_2_0_2_ (30%, 6.3 g) with stirring at room temperature. A precipitate formed at once. Water was removed from the reaction mixture by means of P_2_O_5_ drying in a vacuum oven at room temperature. The solid was washed with three 100 ml portions of ether. The crude product (10 mmol, 1.28 g) was dissolved in 10 ml methanol. Then 20 ml of an ethanol solution of picric acid (10 mmol, 2.29 g) was dropped slowly with stirring, and a yellow precipitate formed at once. The suspension was filtered and dissolved in water. After a few weeks, yellow block crystals were obtained by slow evaporation of the solvent.

### Refinement

Atom H1 was located in adifference Fourier map and refined isotropically. All other H atoms were calculated geometrically and refined using a riding model, with C—H = 0.93-0.97 Å and with *U*_iso_(H) = 1.2 *U*_eq_(C).

### Crystal data

**Table:**

C_6_H_13_N_2_O^+^·C_6_H_2_N_3_O_7_^−^	*F*(000) = 744
*M**_r_* = 357.29	*D*_x_ = 1.550 Mg m^−^^3^
Monoclinic, *P*2_1_/*c*	Mo *K*α radiation, λ = 0.71073 Å
Hall symbol: -P 2ybc	Cell parameters from 5732 reflections
*a* = 11.521 (2) Å	θ = 3.8–27.5°
*b* = 19.230 (4) Å	µ = 0.13 mm^−^^1^
*c* = 6.9921 (14) Å	*T* = 293 K
β = 98.73 (3)°	Prism, yellow
*V* = 1531.2 (5) Å^3^	0.3 × 0.25 × 0.2 mm
*Z* = 4	

### Data collection

**Table:**

Rigaku Mercury2 diffractometer	3495 independent reflections
Radiation source: fine-focus sealed tube	2111 reflections with *I* > 2σ(*I*)
graphite	*R*_int_ = 0.064
CCD Profile fitting scans	θ_max_ = 27.5°, θ_min_ = 3.1°
Absorption correction: multi-scan (*CrystalClear*; Rigaku, 2005)	*h* = −14→14
*T*_min_ = 0.176, *T*_max_ = 0.298	*k* = −24→24
15623 measured reflections	*l* = −9→9

### Refinement

**Table:**

Refinement on *F*^2^	Secondary atom site location: difference Fourier map
Least-squares matrix: full	Hydrogen site location: inferred from neighbouring sites
*R*[*F*^2^ > 2σ(*F*^2^)] = 0.056	H atoms treated by a mixture of independent and constrained refinement
*wR*(*F*^2^) = 0.176	*w* = 1/[σ^2^(*F*_o_^2^) + (0.0905*P*)^2^ + 1.2925*P*] where *P* = (*F*_o_^2^ + 2*F*_c_^2^)/3
*S* = 0.83	(Δ/σ)_max_ < 0.001
3495 reflections	Δρ_max_ = 0.18 e Å^−^^3^
249 parameters	Δρ_min_ = −0.17 e Å^−^^3^
0 restraints	Extinction correction: *SHELXL*, Fc^*^=kFc[1+0.001xFc^2^λ^3^/sin(2θ)]^-1/4^
Primary atom site location: structure-invariant direct methods	Extinction coefficient: 0.018 (2)

### Special details

**Table:**

Geometry. All e.s.d.'s (except the e.s.d. in the dihedral angle between two l.s. planes) are estimated using the full covariance matrix. The cell e.s.d.'s are taken into account individually in the estimation of e.s.d.'s in distances, angles and torsion angles; correlations between e.s.d.'s in cell parameters are only used when they are defined by crystal symmetry. An approximate (isotropic) treatment of cell e.s.d.'s is used for estimating e.s.d.'s involving l.s. planes.
Refinement. Refinement of *F*^2^ against ALL reflections. The weighted *R*-factor *wR* and goodness of fit *S* are based on *F*^2^, conventional *R*-factors *R* are based on *F*, with *F* set to zero for negative *F*^2^. The threshold expression of *F*^2^ > σ(*F*^2^) is used only for calculating *R*-factors(gt) *etc*. and is not relevant to the choice of reflections for refinement. *R*-factors based on *F*^2^ are statistically about twice as large as those based on *F*, and *R*- factors based on ALL data will be even larger.

### Fractional atomic coordinates and isotropic or equivalent isotropic displacement parameters (Å^2^)

**Table:**

	*x*	*y*	*z*	*U*_iso_*/*U*_eq_	Occ. (<1)
C1	0.30226 (19)	0.18175 (13)	0.6927 (3)	0.0374 (5)	
C2	0.3052 (2)	0.25659 (13)	0.7118 (3)	0.0395 (6)	
C3	0.2105 (2)	0.29629 (13)	0.7377 (3)	0.0413 (6)	
H3	0.2160	0.3445	0.7403	0.050*	
C4	0.1062 (2)	0.26361 (13)	0.7599 (3)	0.0392 (6)	
C5	0.0965 (2)	0.19198 (14)	0.7588 (3)	0.0403 (6)	
H5	0.0271	0.1706	0.7794	0.048*	
C6	0.1917 (2)	0.15287 (13)	0.7266 (3)	0.0387 (5)	
C7	0.6393 (2)	0.10824 (17)	0.9188 (4)	0.0584 (8)	
H7A	0.6286	0.0821	1.0335	0.070*	
H7B	0.5852	0.1472	0.9055	0.070*	
C8	0.7660 (3)	0.1343 (2)	0.9351 (6)	0.0926 (13)	
H8A	0.7658	0.1847	0.9319	0.111*	
H8B	0.8091	0.1198	1.0587	0.111*	
C9	0.6332 (2)	0.10287 (15)	0.5683 (4)	0.0510 (7)	
H9A	0.5798	0.1421	0.5511	0.061*	
H9B	0.6177	0.0734	0.4547	0.061*	
C10	0.7612 (3)	0.1282 (2)	0.5971 (6)	0.0763 (10)	
H10A	0.8001	0.1093	0.4951	0.092*	
H10B	0.7619	0.1785	0.5862	0.092*	
C11	0.7006 (2)	0.00311 (16)	0.7672 (5)	0.0630 (8)	
H11A	0.6863	−0.0269	0.6545	0.076*	
H11B	0.6907	−0.0240	0.8806	0.076*	
C12	0.8254 (3)	0.03364 (19)	0.7890 (7)	0.0869 (12)	
H12A	0.8693	0.0186	0.9114	0.104*	
H12B	0.8649	0.0156	0.6864	0.104*	
N1	0.41469 (19)	0.29372 (12)	0.6998 (3)	0.0490 (6)	
N2	0.00623 (19)	0.30586 (13)	0.7894 (3)	0.0510 (6)	
N3	0.1777 (2)	0.07762 (13)	0.7258 (4)	0.0558 (6)	
N4	0.61729 (16)	0.06299 (10)	0.7448 (3)	0.0406 (5)	
N5	0.8256 (2)	0.10837 (14)	0.7815 (4)	0.0641 (7)	
O1	0.50786 (17)	0.26560 (12)	0.7614 (4)	0.0752 (7)	
O2	0.40780 (19)	0.35263 (11)	0.6338 (3)	0.0678 (6)	
O3	0.0149 (2)	0.36879 (12)	0.7812 (4)	0.0798 (7)	
O4	−0.08245 (17)	0.27652 (12)	0.8233 (3)	0.0684 (6)	
O5	0.0766 (4)	0.0514 (3)	0.6742 (11)	0.0852 (18)	0.50
O6	0.2617 (12)	0.0371 (7)	0.7403 (19)	0.066 (2)	0.50
O5'	0.1080 (6)	0.0591 (3)	0.8345 (12)	0.100 (2)	0.50
O6'	0.2427 (13)	0.0432 (7)	0.670 (2)	0.109 (5)	0.50
O7	0.38528 (15)	0.14793 (9)	0.6419 (3)	0.0523 (5)	
O8	0.50320 (16)	0.03390 (10)	0.7282 (3)	0.0545 (5)	
H1	0.457 (3)	0.0704 (19)	0.712 (5)	0.081 (11)*	

### Atomic displacement parameters (Å^2^)

**Table:**

	*U*^11^	*U*^22^	*U*^33^	*U*^12^	*U*^13^	*U*^23^
C1	0.0312 (12)	0.0454 (13)	0.0352 (12)	0.0040 (10)	0.0038 (9)	0.0017 (10)
C2	0.0347 (13)	0.0486 (14)	0.0352 (12)	−0.0032 (10)	0.0054 (10)	−0.0010 (10)
C3	0.0445 (14)	0.0450 (14)	0.0329 (12)	0.0040 (11)	0.0011 (10)	−0.0022 (10)
C4	0.0326 (12)	0.0512 (15)	0.0335 (12)	0.0090 (10)	0.0038 (9)	−0.0019 (10)
C5	0.0312 (12)	0.0570 (15)	0.0329 (12)	0.0011 (11)	0.0055 (9)	0.0020 (11)
C6	0.0351 (12)	0.0457 (14)	0.0356 (12)	0.0018 (10)	0.0068 (9)	0.0032 (10)
C7	0.0515 (17)	0.073 (2)	0.0507 (16)	−0.0038 (14)	0.0083 (13)	−0.0132 (14)
C8	0.061 (2)	0.121 (3)	0.095 (3)	−0.024 (2)	0.0081 (19)	−0.046 (2)
C9	0.0486 (15)	0.0561 (16)	0.0494 (15)	−0.0041 (12)	0.0104 (12)	0.0049 (13)
C10	0.0558 (19)	0.083 (2)	0.094 (3)	−0.0133 (17)	0.0259 (18)	0.020 (2)
C11	0.0495 (17)	0.0467 (16)	0.092 (2)	0.0080 (13)	0.0076 (15)	0.0077 (15)
C12	0.0439 (18)	0.071 (2)	0.144 (4)	0.0117 (16)	0.0082 (19)	0.012 (2)
N1	0.0454 (13)	0.0509 (14)	0.0521 (13)	−0.0051 (10)	0.0120 (10)	−0.0065 (11)
N2	0.0424 (13)	0.0605 (15)	0.0482 (13)	0.0137 (11)	0.0005 (10)	−0.0046 (11)
N3	0.0387 (12)	0.0511 (14)	0.0797 (17)	0.0007 (11)	0.0161 (12)	0.0093 (13)
N4	0.0302 (10)	0.0398 (11)	0.0521 (12)	−0.0012 (8)	0.0069 (8)	0.0013 (9)
N5	0.0356 (12)	0.0671 (17)	0.0889 (19)	−0.0035 (11)	0.0072 (12)	−0.0048 (14)
O1	0.0361 (11)	0.0724 (15)	0.115 (2)	−0.0041 (10)	0.0032 (11)	−0.0038 (13)
O2	0.0672 (14)	0.0541 (13)	0.0866 (16)	−0.0109 (10)	0.0263 (11)	0.0066 (11)
O3	0.0601 (14)	0.0561 (14)	0.123 (2)	0.0194 (11)	0.0128 (13)	−0.0058 (13)
O4	0.0412 (11)	0.0803 (15)	0.0881 (16)	0.0105 (10)	0.0235 (10)	−0.0057 (12)
O5	0.039 (3)	0.054 (3)	0.163 (6)	−0.011 (2)	0.018 (3)	−0.014 (4)
O6	0.046 (3)	0.047 (3)	0.104 (5)	0.004 (2)	0.008 (3)	0.015 (3)
O5'	0.080 (4)	0.072 (4)	0.160 (6)	0.005 (3)	0.059 (4)	0.040 (4)
O6'	0.086 (8)	0.051 (6)	0.204 (15)	−0.005 (5)	0.071 (9)	−0.045 (8)
O7	0.0383 (10)	0.0505 (11)	0.0712 (13)	0.0069 (8)	0.0187 (9)	0.0019 (9)
O8	0.0354 (10)	0.0458 (11)	0.0828 (14)	−0.0078 (8)	0.0108 (9)	0.0026 (10)

### Geometric parameters (Å, °)

**Table:**

C1—O7	1.252 (3)	C10—N5	1.438 (4)
C1—C6	1.442 (3)	C10—H10A	0.9700
C1—C2	1.445 (4)	C10—H10B	0.9700
C2—C3	1.365 (3)	C11—N4	1.492 (3)
C2—N1	1.463 (3)	C11—C12	1.540 (4)
C3—C4	1.386 (3)	C11—H11A	0.9700
C3—H3	0.9300	C11—H11B	0.9700
C4—C5	1.382 (4)	C12—N5	1.438 (4)
C4—N2	1.450 (3)	C12—H12A	0.9700
C5—C6	1.376 (3)	C12—H12B	0.9700
C5—H5	0.9300	N1—O1	1.221 (3)
C6—N3	1.456 (3)	N1—O2	1.221 (3)
C7—N4	1.486 (3)	N2—O3	1.216 (3)
C7—C8	1.531 (4)	N2—O4	1.222 (3)
C7—H7A	0.9700	N3—O6'	1.114 (14)
C7—H7B	0.9700	N3—O6	1.235 (13)
C8—N5	1.447 (5)	N3—O5'	1.239 (6)
C8—H8A	0.9700	N3—O5	1.271 (5)
C8—H8B	0.9700	N4—O8	1.417 (2)
C9—N4	1.488 (3)	O5—O5'	1.134 (8)
C9—C10	1.537 (4)	O6—O6'	0.52 (2)
C9—H9A	0.9700	O8—H1	0.88 (3)
C9—H9B	0.9700		
			
O7—C1—C6	125.4 (2)	N4—C11—C12	107.0 (2)
O7—C1—C2	122.4 (2)	N4—C11—H11A	110.3
C6—C1—C2	112.1 (2)	C12—C11—H11A	110.3
C3—C2—C1	124.1 (2)	N4—C11—H11B	110.3
C3—C2—N1	116.6 (2)	C12—C11—H11B	110.3
C1—C2—N1	119.3 (2)	H11A—C11—H11B	108.6
C2—C3—C4	119.0 (2)	N5—C12—C11	112.6 (3)
C2—C3—H3	120.5	N5—C12—H12A	109.1
C4—C3—H3	120.5	C11—C12—H12A	109.1
C5—C4—C3	121.5 (2)	N5—C12—H12B	109.1
C5—C4—N2	119.6 (2)	C11—C12—H12B	109.1
C3—C4—N2	118.9 (2)	H12A—C12—H12B	107.8
C6—C5—C4	118.7 (2)	O1—N1—O2	123.2 (2)
C6—C5—H5	120.7	O1—N1—C2	118.9 (2)
C4—C5—H5	120.7	O2—N1—C2	117.8 (2)
C5—C6—C1	124.2 (2)	O3—N2—O4	123.1 (2)
C5—C6—N3	117.0 (2)	O3—N2—C4	118.6 (2)
C1—C6—N3	118.8 (2)	O4—N2—C4	118.3 (2)
N4—C7—C8	107.1 (2)	O6'—N3—O5'	125.3 (8)
N4—C7—H7A	110.3	O6—N3—O5'	110.0 (7)
C8—C7—H7A	110.3	O6'—N3—O5	107.5 (9)
N4—C7—H7B	110.3	O6—N3—O5	116.5 (8)
C8—C7—H7B	110.3	O5'—N3—O5	53.7 (4)
H7A—C7—H7B	108.5	O6'—N3—C6	120.9 (8)
N5—C8—C7	112.7 (3)	O6—N3—C6	122.9 (7)
N5—C8—H8A	109.0	O5'—N3—C6	111.4 (4)
C7—C8—H8A	109.0	O5—N3—C6	119.4 (3)
N5—C8—H8B	109.0	O8—N4—C7	109.82 (19)
C7—C8—H8B	109.0	O8—N4—C9	111.43 (18)
H8A—C8—H8B	107.8	C7—N4—C9	110.5 (2)
N4—C9—C10	106.9 (2)	O8—N4—C11	106.12 (19)
N4—C9—H9A	110.3	C7—N4—C11	109.8 (2)
C10—C9—H9A	110.3	C9—N4—C11	109.0 (2)
N4—C9—H9B	110.3	C12—N5—C10	107.1 (3)
C10—C9—H9B	110.3	C12—N5—C8	108.3 (3)
H9A—C9—H9B	108.6	C10—N5—C8	109.6 (3)
N5—C10—C9	112.8 (2)	O5'—O5—N3	61.7 (4)
N5—C10—H10A	109.0	O6'—O6—N3	64 (3)
C9—C10—H10A	109.0	O5—O5'—N3	64.6 (4)
N5—C10—H10B	109.0	O6—O6'—N3	91 (3)
C9—C10—H10B	109.0	N4—O8—H1	103 (2)
H10A—C10—H10B	107.8		

### Hydrogen-bond geometry (Å, °)

**Table:**

*D*—H···*A*	*D*—H	H···*A*	*D*···*A*	*D*—H···*A*
O8—H1···O7	0.88 (3)	1.74 (4)	2.602 (3)	167 (3)
O8—H1···O6'	0.88 (3)	2.50 (4)	2.973 (15)	115 (3)
C7—H7B···O1	0.97	2.59	3.486 (4)	153
C9—H9A···O7	0.97	2.42	3.102 (3)	127
C5—H5···N5^i^	0.93	2.61	3.536 (3)	171
C11—H11A···O6'^ii^	0.97	2.54	3.342 (15)	139
C12—H12A···O5'^iii^	0.97	2.31	3.176 (9)	149
C12—H12B···O5^iv^	0.97	2.55	3.137 (7)	119

Symmetry codes: (i) *x*−1, *y*, *z*; (ii) −*x*+1, −*y*, −*z*+1; (iii) −*x*+1, −*y*, −*z*+2; (iv) *x*+1, *y*, *z*.
